# Branched-Chain Amino Acids in Liver Diseases: Complexity and Controversy

**DOI:** 10.3390/nu16121875

**Published:** 2024-06-14

**Authors:** Yaqi Zhang, Luqi Zhan, Lingjian Zhang, Qingmiao Shi, Lanjuan Li

**Affiliations:** 1 State Key Laboratory for Diagnosis and Treatment of Infectious Diseases, National Clinical Research Center for Infectious Diseases, National Medical Center for Infectious Diseases, Collaborative Innovation Center for Diagnosis and Treatment of Infectious Diseases, The First Affiliated Hospital, Zhejiang University School of Medicine, 79 Qingchun Rd., Hangzhou 310003, China; 2 Affiliated Hangzhou First People’s Hospital, School of Medicine, Westlake University, Hangzhou 310024, China

**Keywords:** branched-chain amino acids, liver disease, mTOR, nutrition, supplements

## Abstract

Branched-chain amino acids (BCAAs), as essential amino acids, engage in various physiological processes, such as protein synthesis, energy supply, and cellular signaling. The liver is a crucial site for BCAA metabolism, linking the changes in BCAA homeostasis with the pathogenesis of a variety of liver diseases and their complications. Peripheral circulating BCAA levels show complex trends in different liver diseases. This review delineates the alterations of BCAAs in conditions including non-alcoholic fatty liver disease, hepatocellular carcinoma, cirrhosis, hepatic encephalopathy, hepatitis C virus infection, and acute liver failure, as well as the potential mechanisms underlying these changes. A significant amount of clinical research has utilized BCAA supplements in the treatment of patients with cirrhosis and liver cancer. However, the efficacy of BCAA supplementation in clinical practice remains uncertain and controversial due to the heterogeneity of studies. This review delves into the complicated relationship between BCAAs and liver diseases and tries to untangle what role BCAAs play in the occurrence, development, and outcomes of liver diseases.

## 1. Introduction

Branched-chain amino acids (BCAAs) refer to proteinogenic amino acids that have aliphatic branched side chains in their chemical structure, consisting of valine (Val), leucine (Leu), and isoleucine (Ile). As key components of most proteins, BCAAs can be synthesized by bacteria, plants, and fungi but not by metazoans, which makes them essential amino acids for the human body [1]. The main catabolic pathways of the three BCAAs are the same, and the molar relative abundance between them is almost always about 1.6:2.2:1.0 (Val:Leu:Ile) [2]. They are mostly used in the form of mixtures in practical applications, thus generally studied as a whole. In addition to representing an indispensable nutrient, BCAAs and their many metabolites also function as signaling molecules, with the most extensively studied area being their regulatory effect on the mechanistic target of rapamycin (mTOR) pathway [3].

The liver holds a vital position in BCAA oxidative degradation and incorporation into protein [4]. Fischer’s ratio, the molar ratio of plasma BCAAs to aromatic amino acids (AAAs), was first proposed in 1971 [5], and many studies have validated its significance in evaluating liver metabolism, liver functional capacity, and the degree of liver impairment. A low Fischer’s ratio is a hallmark of liver cirrhosis and promotes hepatic encephalopathy (HE) [6,7]. Multiple metabolomic studies conducted recently have demonstrated a correlation between alterations in circulating BCAA levels and a variety of liver diseases [8,9,10]. It has been suggested that BCAA supplementation favors anabolic pathways and thereby has a promising effect in reducing cachexia, preventing or treating symptoms of HE, and ameliorating liver disease [11,12,13]. However, the use of BCAAs in nutritional supplements remains controversial, and no consensus has been reached. This review intends to explore the relationship between the liver and BCAA metabolism, assess the causes of alterations in BCAA levels in various liver diseases, and provide updated perspectives on their use in nutritional supplements for major possible liver disease indications.

## 2. Metabolism and Signal Transduction in BCAAs in the Liver

The distribution of BCAA in the human body can be conceptualized as a two-compartment model: a circulation pool and a tissue pool [2]. BCAAs in the circulating pool primarily originate from dietary intake and protein breakdown. When entering the tissue pool, BCAAs are oxidized and removed or used to synthesize new proteins. The catabolic pathway of BCAAs involves two main common steps: reversible transamination mediated by branched-chain aminotransferase (BCAT) and irreversible decarboxylation catalyzed by the branched-chain α-keto acid dehydrogenase (BCKDH) complex [14]. Various tissues are involved in regulating BCAA decomposition, with the liver and muscles playing pivotal roles. Due to the relatively low hepatic activity of BCAT, only a small proportion of the initial metabolic reaction of BCAAs occurs in the liver. The majority is taken up by skeletal muscles after entering systemic circulation through the liver [15]. The reversible transfer of the amino group of BCAAs to α-ketoglutarate (α-KG) results in the formation of glutamate and the corresponding branched-chain keto acids (BCKAs). In contrast to BCAT, the second BCAA catabolic enzyme, BCKDH, a multienzyme complex situated on the inner surface of the inner mitochondrial membrane, exhibits the highest activity in the liver [16], indicating that a significant portion of BCKAs is released from the muscle into the circulation pool and subsequently returned to the liver for further metabolism. Research has shown that brain insulin signaling regulates BCAA metabolism by inducing the expression and activity of hepatic BCKDH [17]. Reduced hepatic BCKDH is a primary cause of increased plasma BCAA levels, potentially explaining the elevated plasma BCAA levels in insulin-resistant patients. BCKDH catalyzes the decarboxylation of BCKAs to generate the respective branched-chain acyl-CoA esters and release CO_2_. The third step of BCAA catabolism occurs within the mitochondrial matrix, where each BCAA enters the tricarboxylic acid (TCA) cycle through its own pathway to produce ATP. Depending on the number of carbon atoms entering the TCA cycle, Val is considered glucogenic, Leu is ketogenic, and Ile can serve as both. Under homeostatic conditions, circulating BCAA levels remain stable, maintaining a balance between intake and loss [18]. Changes in circulating concentrations of BCAAs are associated with a plethora of liver diseases. Fischer’s ratio and a simplified version known as the BCAA-to-tyrosine ratio (BTR) are recognized as valuable indicators of amino acid imbalance and pathophysiological disorders in liver diseases [19,20]. With the progression of liver dysfunction, there is a decrease in plasma BCAA levels and an increase in AAA levels, leading to a reduction in Fischer’s ratio.

**Figure 1:**
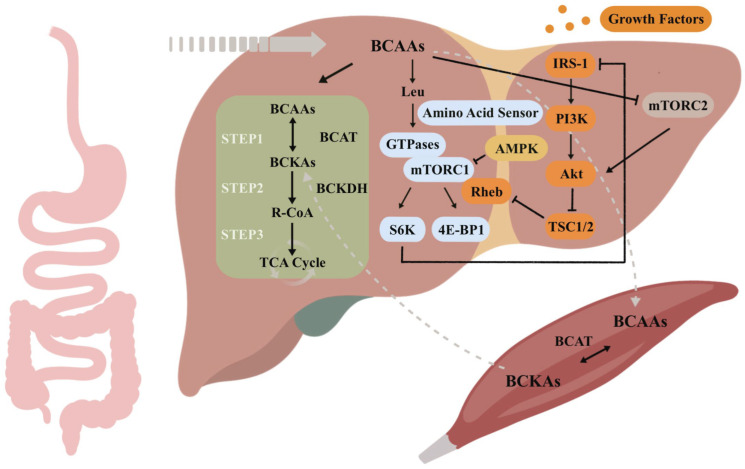
Schematic representation of BCAA metabolism in the liver and the signal transduction processes involved. BCAAs, branched-chain amino acids; BCAT, branched-chain aminotransferase; BCKAs, branched-chain keto acids; BCKDH, branched-chain α-keto acid dehydrogenase; R-CoA, respective branched-chain acyl-CoA esters; TCA, tricarboxylic acid; Leu, leucine; GTPases, guanosine triphosphatases; mTORC1, mTOR complex 1; Rheb, Ras homolog enriched in brain; AMPK, AMP-activated protein kinase; S6K, ribosomal protein S6 kinase; 4E-BP1, eukaryotic translation initiation factor 4E-binding protein 1; IRS-1, insulin receptor substrate-1; PI3K, phosphatidylinositol-4,5-bisphosphate 3-kinase; Akt, protein kinase B; TSC, tuberous sclerosis; mTORC2, mTOR complex 2.

Besides their role as nutrients, BCAAs also serve a crucial function in allosteric regulation and signal transduction (Figure 1). When the human body is in different energy states, BCAAs can activate distinct signaling pathways to promote metabolic adaptations to uphold energy homeostasis, among which the modulation of the mTOR pathway is the most extensively studied. mTOR comprises two disparate complexes, known as mTOR complex 1 (mTORC1) and mTOR complex 2 (mTORC2), each receiving unique upstream inputs and yielding separate downstream outputs. mTORC1 is regulated by five extracellular and intracellular signals—growth factors [such as insulin and insulin-like growth factor 1 (IGF-1)], cellular energy levels (high ATP/AMP ratio), amino acids, stress, and oxygen—to promote anabolism, including protein translation and lipid synthesis and to inhibit catabolism, such as autophagy, thereby shifting metabolic states [21,22]. The process through which BCAAs activate mTORC1 is called nutritive sensing, but our understanding of the molecular mechanisms by which mTORC1 senses intracellular amino acids is still evolving. A 2008 study by Sancak et al. uncovered that Rag proteins, four related guanosine triphosphatases (GTPases) that form obligate heterodimers consisting of RagA/B with RagC/D, interact with mTORC1 in an amino-acid-sensitive manner [23]. This interaction propels mTORC1 translocation to lysosomal membranes that contain the mTORC1 activator known as Ras homolog enriched in brain (Rheb) [24]. Rheb binds to mTORC1 and activates its kinase in a growth-factor-dependent manner [25], so the amino-acid-regulated recruitment of mTORC1 is a prerequisite for growth factor activation of mTORC1. While mTORC1 activity is significantly influenced by alterations in amino acid levels, it does not function as an amino acid sensor. A 2016 study by Wolfson et al. found that Sestrin2 is a Leu sensor for the mTORC1 pathway [3]. Leu triggers mTORC1 activation by interacting with Sestrin2, a protein that binds to GATOR2 and suppresses mTORC1 signaling, leading to the release of GATOR2, a positive mTORC1 regulator. It has also been shown that leucyl-tRNA synthetase (LRS) initiates molecular events that lead to mTORC1 activation by sensing the concentration of Leu in cells [26,27,28]. Growth factors and cellular energy regulate mTORC1 through the tuberous sclerosis (TSC) 1/2 heterodimer, in a manner different from amino acids. Under conditions of low cellular energy (high AMP/ATP ratio), AMP-activated protein kinase (AMPK) inhibits mTOR’s function and triggers the catabolic pathway of energy production. In instances of amino acid deficiency, AMPK enhances the intracellular uptake of BCAAs and fosters oxidation through various hormones [29].

Activated mTORC1 phosphorylates the downstream translation initiation factors known as eukaryotic translation initiation factor 4E-binding protein 1 (4E-BP1) and ribosomal protein S6 kinase (S6K) to promote ribosome biogenesis and mRNA translation, thereby regulating protein synthesis [30]. However, the persistent activation of mTORC1 by BCAAs leads to insulin resistance (IR), attributed to mTORC1-S6K-mediated negative feedback loops that shut down the downstream cascades dependent on insulin. TSC1/2 acts as a GTPase-activating protein (GAP) for Rheb GTPase, inhibiting mTORC1 activity by converting Rheb into its inactive GDP-bound state [31]. The binding of insulin to receptor tyrosine kinases (RTKs) phosphorylates insulin receptor substrate-1 (IRS-1), subsequently activating phosphatidylinositol-4,5-bisphosphate 3-kinase (PI3K), which, in turn, recruits and activates protein kinase B (Akt/PKB) [32,33]. Akt phosphorylates TSC2 to deactivate the TSC complex, therefore facilitating mTORC1 regulation through the IRS-PI3K-AKT axis [34]. Upon activation, mTORC1 and S6K directly phosphorylate IRS-1 to induce its degradation and alter its localization [35,36], thereby dampening PI3K/Akt activation and ultimately negatively regulating growth factor signaling. A study conducted in liver cancer cells verified that BCAAs also inhibit the PI3K/Akt pathway by suppressing mTORC2 kinase activity on Akt, suggesting that supplemental BCAAs may impede liver cancer progression by inhibiting insulin-induced PI3K/Akt signaling and subsequent anti-apoptotic pathways [37].

## 3. Association of BCAAs with Liver Diseases

### 3.1. Non-Alcoholic Fatty Liver Disease

The prevalence of non-alcoholic fatty liver disease (NAFLD) is increasing and can be as high as 30% in developed countries. NAFLD is characterized by macrovesicular steatosis in ≥5% of hepatocytes in individuals who consume minimal-to-no alcohol, frequently accompanying obesity, type 2 diabetes (T2D), and IR [38]. In 2023, NAFLD was officially renamed as metabolic-dysfunction-associated steatotic liver disease (MASLD), referring to patients with hepatic steatosis and at least one of five cardiometabolic risk factors, emphasizing the significant increase in liver fibrosis and cirrhosis risk associated with cardiometabolic disorders [39].

The increase in plasma BCAA concentration is one of the important manifestations of the NAFLD metabolic profile [40]. Metabolomic analysis of obese and lean individuals indicated that, in the context of overnutrition and low IGF-1 levels among obese subjects, circulating BCAA overload results in sustained activation of the mTOR/S6K pathway, contributing to IR and an increased BCAA catabolic flux, which ultimately leads to elevated gluconeogenesis and glucose intolerance [41]. Elevated plasma BCAAs are inversely correlated with insulin sensitivity and the metabolic clearance of insulin while showing a positive association with fasting insulin levels [42], which may be related to the insulinotropic effects of Leu and Ile [43].

BCAAs could be an important link between the development and progression of obesity, IR, NAFLD, and T2D. In patients without diabetes but diagnosed with NAFLD through biopsy, obese individuals exhibit higher plasma levels of BCAAs compared to non-obese individuals. Furthermore, the presence of balloon-like changes and/or inflammation during liver biopsy is associated with elevated plasma BCAAs [44]. In a large prospective study primarily involving individuals of Caucasian descent from the general population, data analysis of 5791 participants without T2D at baseline assessment revealed elevated fasting total plasma BCAA levels in subjects with NAFLD. This association remained unaffected by various clinical and laboratory covariates, encompassing assessments of IR and β-cell function. During a median follow-up period of 7.3 years, a total of 276 participants developed T2D, with 70.2% of them exhibiting elevated fatty liver index levels, indicating a significantly increased risk of developing T2D in individuals with elevated FLI. The predictive value of higher FLI for the advancement of T2D is partly attributed to elevatory plasma BCAA levels, suggesting a potential combined effect of NAFLD and elevated circulating BCAAs on T2D [40].

There are conflicting statements regarding whether the elevation of peripheral BCAA levels in NAFLD patients is dependent on sex. A study published in 2015 involving 101 individuals aged 50 to 55 showed that elevated serum BCAA levels are associated with early stage liver fat accumulation, independent of gender, obesity, or IR [45]. Another cross-sectional cohort study that enrolled 112 obese patients from 2006 to 2014 observed a significant effect of gender on the association between plasma BCAA concentration and NAFLD severity. Sex-specific subgroup analysis emphasized a pattern wherein plasma BCAA levels rose with NAFLD severity in females but declined in males. Furthermore, only females showed an increase in liver fibrosis degree with a rise in plasma BCAAs [46].

In order to change the current situation of liver biopsy still being the “gold standard” for the diagnosis of hepatic steatosis, attempts have been made to develop non-invasive predictive models based on the characteristics of NAFLD metabolic disorders. A study conducted in severely obese children and adolescents, with the aim of investigating novel biomarkers for NAFLD has developed a BCAA-based metabolic score that can predict the degree of hepatic steatosis in high-risk pediatric populations [9].

### 3.2. Hepatocellular Carcinoma

Diabetes is a risk factor for hepatocellular carcinoma (HCC) [47,48,49], and its presence alongside chronic liver disease is not only closely associated with the progression of HCC but also linked to a poor prognosis [50]. Further studies showed that hyperinsulinemia based on underlying chronic liver disease promoted the growth of human HCC and affected its early clinical advancement [51,52]. A multicenter, randomized, controlled trial involving 622 patients with decompensated cirrhosis found that long-term oral BCAA supplementation inhibited the evolution of liver cancer in obese patients [53]. In obese diabetic rats, BCAA exerts a chemopreventive effect on HCC by inhibiting vascular endothelial growth factor (VEGF) expression and hepatic neovascularization [54]. Subsequent cellular-level studies revealed that BCAA primarily modulates VEGF expression post-transcriptionally, necessitating all three BCAA components to expedite insulin-induced VEGF mRNA degradation [55]. The transition of cancer stem cells (CSCs) to cancer cells heightens their responsiveness to chemotherapy drugs. BCAAs can activate mTORC1, while the activated mTORC1 inhibits mTORC2, synergistically reducing the number of CSCs and enhancing chemotherapy sensitivity [56].

A plasma metabolomics study based on gas chromatography–mass spectrometry (GC-MS) identified elevated BCAA levels in HCC patients [10]. Another study, however, came to a different conclusion. A retrospective analysis of 1270 patients diagnosed with liver cancer between 2008 and 2020 revealed that a low BTR (≤4.4) could serve as a valuable prognostic indicator in chronic liver disease (CLD) patients with early HCC. Additionally, there was a significant negative correlation between BCAA levels and albumin–bilirubin scores [57]. This apparent discrepancy in findings could stem from variations in etiology and sample sizes between the two studies. The former study recruited 40 HCC patients in Egypt, all with hepatitis C virus (HCV) infection and without hepatitis B virus (HBV) infection, while the latter study in Japan encompassed a larger sample size and a more diverse etiology, with HCV infection only constituting 60% of the cases. Another recent analysis of the amino acid profile of the Egyptian population, involving 302 participants, showed that the infected group (HCC, HBV, HCV, and co-infected patients) exhibited decreased levels of BCAAs, increased levels of AAAs, and significant changes in Fisher ratio and BTR compared with the healthy control group [58].

The changes in BCAA in HCC patients are not only manifested in the circulating level but also in the metabolism of cancer and adjacent tissues. An analysis of 26 pairs of severely fibrotic or cirrhotic human HCC and adjacent non-tumor tissues demonstrated the accumulation of BCAAs in the HCC tissues [59]. A study employing a multi-omics approach in animal models and HCC patients implied that BCAA catabolism was disrupted during tumor initiation and progression but remained intact in normally proliferating cells, suggesting that the loss of BCAA catabolism fosters tumor initiation and growth [60]. The accumulation of BCAAs in liver cancer tissues may also contribute to their changes in circulating levels. 

The conflicting needs of the tumor and the host complicate the normal requirement for BCAAs. Heightened BCAA levels correlated with increased visceral fat mass and served as prognostic markers for enhanced overall survival in HCC patients [61]. The differences in BCAA level alterations between peripheral circulation and HCC tissues could be due to their different uses: circulating BCAAs are utilized to sustain energy metabolism, whereas cancer cells exploit them to activate oncogenic signaling pathways. 

### 3.3. Cirrhosis

The changes in plasma amino acid profiles in patients with liver cirrhosis showed a decreased Fisher’s ratio due to the diminished BCAA content and raised AAA levels, and this change was positively correlated with the level of albumin [62]. Protein–energy malnutrition (PEM) is a common clinical manifestation in cirrhosis patients, with reported incidence rates exceeding 65% [63]. Protein malnutrition in cirrhosis is characterized by reduced serum albumin level and decreased skeletal muscle mass. The reduction in BCAA levels in cirrhosis is closely linked to the deterioration of protein malnutrition and HE. Patients with cirrhosis were found to have a lower relative clearance of BCAAs, and this alteration was significantly correlated with elevated blood ammonia levels [64]. Due to cirrhosis causing an impaired hepatic ammonia detoxification capacity, leading to elevated blood ammonia levels, the skeletal muscle will uptake BCAAs as precursors to synthesize glutamine, subsequently incorporating ammonia during the process of glutamine-generating glutamine synthetase to eliminate blood ammonia. Moreover, the augmented uptake of BCAAs by skeletal muscles is also intricately linked to shifts in thermogenic characteristics in cirrhosis patients. While glucose typically serves as the paramount energy substrate for thermogenesis under physiological circumstances, cirrhotic individuals, afflicted by hepatic atrophy, experience the depletion of hepatic glycogen reserves and exhibit IR in peripheral tissues. Consequently, under conditions of liver cirrhosis, the energy efficacy of glucose markedly diminishes, juxtaposed with a physiological surge in the energy efficiency of BCAAs from 45% to 96% [65]. Compared with AAAs that are exclusively metabolized in the liver, BCAAs are mainly metabolized in peripheral tissues represented by skeletal muscles, which is the reason for their opposite change trend. The transamination reaction of BCAAs to produce BCKA and glutamate is reversible, and many studies have illustrated the liver’s ability to reaminate BCKA and release the corresponding BCAAs back into the bloodstream. The primary reactions of BCAA metabolism in the liver occur in mitochondria, and the impairment of mitochondrial function has been observed in various chronic liver diseases [66]. Therefore, the weakened reamination of BCAAs due to hepatic mitochondrial abnormality may be an additional mechanism contributing to the reduced BCAA levels in cirrhotic patients.

### 3.4. Hepatic Encephalopathy

HE is a reversible syndrome characterized by a range of neuropsychiatric abnormalities that manifest in patients with advanced liver dysfunction following the exclusion of other established brain diseases. The elevated blood ammonia concentration resulting from impaired liver function has long been considered the primary cause of HE. The false neurotransmitter hypothesis, proposed by Fischer and Baldessarini in the 1980s, shifts focus to the interplay between amino acids and neurotransmission [67]. Physiologically, both AAAs and BCAAs are large neutral amino acids (LNAAs) that make use of a shared transport system to cross the blood–brain barrier. Since all LNAAs must compete with each other for transport across the blood–brain barrier, their relative concentrations are more critical than their absolute concentrations in determining brain uptake. Therefore, the transport of AAAs to the brain can increase not only through an absolute increase in plasma concentration but also through a decrease in plasma BCAA levels. A low Fischer’s ratio leads to AAAs mass entry into the central nervous system, potentially causing an imbalance in the synthesis of dopamine, norepinephrine, and serotonin. This can result in an increase in false neurotransmitter (dopamine, norepinephrine) production, which inhibits the synthesis of normal neurotransmitters and competes with them, enhancing inhibitory neural activity.

Fischer’s ratio exhibits a robust correlation with the grading of HE, and an improvement in HE is noted upon the normalization of this ratio to within the standard range. Fischer’s solution, enriched with BCAAs, was utilized for HE treatment as early as 1976 [68]. However, others believe that amino acid imbalance is not the cause of HE, but is more likely to be the result of ammonia poisoning-induced reduction in BCAA levels after liver damage.

### 3.5. Hepatitis C Virus Infection

Infection with HCV has the potential to result in the progression of chronic liver disease, ultimately culminating in cirrhosis and chronic liver failure. BCAAs, notably Val, influence the function of human monocyte-derived dendritic cells (MoDCs). CD14-positive monocytes isolated from peripheral blood mononuclear cells (PBMCs) from both healthy volunteers and patients with HCV cirrhosis presented an inability to differentiate into mature dendritic cells in a BCAA- or Val-deficient medium, displaying low CD83 expression. Val dose-dependently boosted the isostimulatory capacity and interleukin production of MoDCs from both healthy volunteers and HCV cirrhosis patients. Elevated extracellular Val levels have been noted to improve dendritic cell function in cirrhotic patients [69]. Among individuals with advanced cirrhosis, administering BCAAs orally can stimulate peripheral blood monocytes in autologous plasma, resulting in a marked increase in interferon (IFN)-γ production [70]. A case report documented a patient with HCV-related decompensated cirrhosis who was treated with an oral Val agent, resulting in a significant decrease in HCV RNA levels. Subsequently, the patient underwent IFN-β therapy, successfully eradicating chronic HCV infection [71]. In vitro cellular studies have found that BCAA has two opposite effects on HCV production: the inhibition of HCV genomic RNA replication and the promotion of infectious virus formation in infected cells. BCAAs can dose-dependently inhibit the HCV replicon, with this effect being mTOR pathway-independent. Val was pinpointed as the key contributor [72]. A study of plasma amino acid profiles and hepatic gene expression in 168 patients with advanced chronic hepatitis C undergoing treatment with a combination of pegylated IFN and ribavirin found that Fischer’s ratio was significantly associated with nonresponders and influenced by hepatic mTORC1 signaling. Cellular studies proved that malnutrition hinders IFN signaling by suppressing mTORC1 and triggering Socs3 signaling via Foxo3a. Elevating BCAA levels to enhance IFN signaling could emerge as a novel approach for individuals with advanced chronic hepatitis C [73]. Another retrospective study on the effect of BCAA supplementation on IFN therapy in Japanese patients with chronic HCV infection also showed consistent results [74].

### 3.6. Acute Liver Failure

The decreased levels of BCAAs resulting from various complex factors in chronic liver disease persist into the decompensated stage of chronic liver failure (CLF). However, during acute liver failure (ALF), changes in BCAA levels show varying trends: decreases, similarity, and increases have been reported. A study using rat models of acute and chronic liver failure found that ALF was characterized by increased plasma BCAAs and BCKA levels and decreased hepatic BCKDH activity, whereas CLF was characterized by decreased plasma BCAA and BCKA levels and increased hepatic BCKDH activity [75]. BCAA catabolism is inhibited in ALF and heightened in CLF, resulting in varied outcomes from oral BCAA supplementation. Administering BCAAs in ALF may result in nitrogen overload, hastening the onset of hyperammonemia and hepatic encephalopathy. While numerous studies in rat models have validated the protective role of BCAAs against acute liver injury [76,77,78] and their positive influence on liver regeneration [79,80,81], their effects on humans remain uncertain.

## 4. Clinical Application of BCAAs in the Treatment of Liver Diseases

In Japan, clinical studies on BCAA supplementation for patients with liver disease have been conducted since the 1980s [82]. The practice guideline published in 2014 by the European Association for the Study of the Liver (EASL) and the American Association for the Study of Liver Diseases (AASLD) recommends an oral BCAA-enriched nutritional formulation for the treatment of HE and for improving the nutritional status of cirrhotic patients [83]. The clinical nutrition guidelines of the European Society of Parenteral and Enteral Nutrition (ESPEN) [84] also make the same recommendation. Many nutritional supplements containing different doses of BCAAs have been developed and commercialized over the years, but two prominent products, Aminoleban^®^ EN (Otsuka Pharmaceutical Co. Ltd., Tokyo, Japan), launched in 1988, and LIVACT^®^ Granules (Ajinomoto Co. Inc., Tokyo, Japan), launched in 1996, have been widely used in liver disease patients and have been extensively reported on in the literature.

Table 1 summarizes clinical trials applying BCAA supplements for the treatment of HCC, unequivocally showcasing the substantial influence of BCAAs on the optimization of diverse facets of patient outcomes. Studies conducted over the years have consistently shown favorable effects of BCAAs in HCC patients after hepatectomy and after radiofrequency ablation (RFA). BCAA administration in HCC patients undergoing transcatheter arterial chemoembolization (TACE) prevented treatment-induced liver function suppression [85]. Impaired hepatic synthetic function results in decreased levels of total protein and albumin, while BCAAs aid in increasing and maintaining albumin levels [86,87,88,89]. In HCC patients with concomitant cirrhosis, the consumption of BCAA-rich supplements caused an improvement in the Child–Pugh score [90]. Specifically, BCAAs exhibited a synergistic effect in anti-angiogenesis and ameliorating IR in individuals already afflicted with it [91]. Moreover, BCAAs have been associated with shorter hospital stays and improved quality of life (QOL) for patients undergoing different treatment modalities [92,93,94]. A 26-week prospective study found that administering BCAAs could lower the incidence of early recurrence [95], whereas the fact that prolonged BCAA supplementation might reduce the likelihood of intrahepatic recurrence and associated complications was observed in another 5-year randomized trial [96]. Despite not all studies confirming a reduction in the risk of recurrence, BCAAs provide noteworthy benefits for specific patient populations, such as younger individuals with mild impairments in glucose tolerance [97]. The synergistic use of BCAAs alongside other interventions, including angiotensin-converting enzyme inhibitor (ACEI) or radiofrequency ablation, has yielded promising results in suppressing tumor recurrence and strengthening biochemical profiles [98,99]. Patients with HCC who have normal albumin levels but low BTR can benefit from BCAA therapy in terms of both overall and disease-specific survival rates [100], while its therapeutic efficacy in other populations of HCC patients remains to be further investigated. These findings suggest that BCAAs have a multifaceted impact on the treatment of HCC, providing a range of benefits that contribute to better patient outcomes and potentially improve survival rates in the long term.

Table 2 outlines the clinical research on the applications of BCAAs in the management of cirrhosis. After the utilization of BCAAs, advancements in serum albumin level were noticed in patients with cirrhosis, whether in the early stage, in the decompensated stage, or complicated by HE [102,103,104,105]. A randomized study comparing the effects of BCAA supplementation on serum albumin levels in compensated and decompensated cirrhosis discovered that patients in a compensated state, despite low BTR, were able to maintain plasma albumin levels for two years with BCAA intake [106]. Oral supplementation with BCAAs may mitigate the risk of HCC in cirrhotic patients with certain characteristics. A multicenter, randomized, controlled trial comprising 622 individuals with decompensated cirrhosis reported a remarkable decrease in liver cancer risk among those in the long-term oral BCAA group with a BMI ≥ 25 and AFP levels ≥ 20 ng/mL [53]. It has also been researched that BCAAs may inhibit hepatocarcinogenesis in individuals with compensated hepatitis C cirrhosis and serum albumin levels < 4.0 g/dL [107]. For those with advanced cirrhosis, BCAAs exhibit promising outcomes in preventing progressive hepatic failure and improving surrogate markers, as well as improving perceived health status [108]. Of particular importance to patients waiting for liver transplantation, early oral BCAA intervention could preserve liver reserves and extend the waiting period [109]. However, a recent study in North American HCV-infected patients with advanced fibrosis or compensated cirrhosis did not find an association between dietary BCAA intake and liver-related outcomes [110]. The potency of BCAAs in alleviating HE and sarcopenia, two prevalent complications of cirrhosis, has also been thoroughly explored. In cases of HE, BCAAs were found to improve minimal HE symptoms while not lessening the recurrence [111]. Patients with cirrhosis frequently suffer from sarcopenia, a condition defined by lowered muscular mass, quality, and strength that is linked to poor clinical outcomes. Taking BCAAs at night could reduce the occurrence of leg muscle cramps, and taking them for a months or more could improve muscle mass [112,113,114,115,116]. The mechanism behind this may be that impaired mTOR1 signaling and increased autophagy in skeletal muscle are reversed by BCAAs [117]. However, there is also a study that came to a negative conclusion [118,119].

Another focal point in clinical applications is the timing of BCAA supplementation within the day. Given that patients with liver cirrhosis prioritize BCAAs as energy substrates [65], the timing of BCAA intake becomes crucial. If taken during the daytime, the majority of BCAAs may be utilized for energy production rather than protein synthesis. Late-evening snacks (LESs) enriched with BCAA can offer additional protein and calories before bedtime, assisting in curbing fat oxidation and preventing the onset of a catabolic state after overnight fasting. Taking BCAAs before bedtime was more beneficial for patients than consuming regular carbohydrates as LESs [105]. However, patients with cirrhosis often have abnormal glucose metabolism, making the impact of nocturnal supplementation with BCAA on glucose metabolism particularly noteworthy. A number of studies have shown that providing BCAA-enriched LESs can ameliorate energy malnutrition and glucose tolerance in patients with cirrhosis or HCC [101,120,122,123,125]. Nonetheless, a pilot study in 2019 found that obesity and severe IR may be potential risk factors for deteriorating glucose homeostasis in cirrhotic patients receiving BCAA-enriched LESs [127]. The discrepancies in study outcomes may be attributed to variations in the duration of BCAA administration and the hepatic functional reserves of participants. In general, the nocturnal administration of BCAA-enriched LESs is usually safe for cirrhotic patients, with no adverse effects, and shows better results than equal doses taken during the day [121,126]. Still, it may be more suitable for those without obesity and IR.

In clinical trials like these, the key to establishing a control is to provide equal amounts of energy and protein to all groups. Researchers typically provide a specialized isonitrogenous isocaloric diet to the control group, equivalent to the BCAA intervention group. However, this approach has obvious limitations; it necessitates high patient compliance and requires researchers to provide adequate nutritional guidance to patients. Ideally, having nutritionists track patients throughout the study period is optimal, to ensure the implementation of this dietary regimen. Patients may also receive treatment for underlying liver diseases during the trial, which also could influence the outcomes. The trial protocol’s implementation challenges and the study population’s unique characteristics both possibly increase the risk of potential biases in the trial.

We identified a blind spot that was easily overlooked in the initial experimental design: the protein and energy requirements of patients with liver disease. According to the evidence-based clinical nutrition guidelines for liver disease [84], it is recommended that patients with chronic liver disease and a sedentary lifestyle receive a total energy supply of 1.3 times resting energy expenditure (REE) (24 kcal/kg/day), which means that their energy requirement is 32 kcal/kg/day. Patients with compensated cirrhosis who are not malnourished should ingest 1.2 g/kg/d protein, whereas those with manifestations of malnutrition and/or sarcopenia should ingest 1.5 g/kg/d·protein.

Only a very small number of trials emphasize that the BCAA supplement provided, when combined with the patients’ daily diet, could meet the international recommended standards of 30–35 kcal/kg/d of energy and 1.2–1.5 g/kg/d of protein for patients with liver disease. Most trials simply provided diets with equal protein and energy for all enrolled patients without accounting for weight considerations, and they did not specify whether the protein and energy provided met the baseline requirements of the patients. In many clinical trials involving patients with HCC who had undergone liver resection or were undergoing radiotherapy or chemotherapy, the energy and protein requirements under these circumstances were definitely different. It is evident that the supplementation of BCAAs under the conditions of meeting or not meeting the protein and energy requirements of patients represents two entirely different scenarios.

We speculate that this may be a key reason why, despite numerous clinical trials conducted over the past few decades, a consensus has not been reached on the therapeutic efficacy of BCAA supplementation in patients with liver diseases. Addressing this issue involves refining patient stratification in experimental design, taking into account not only weight but also specific disease stages and comorbidities such as diabetes or renal failure, specifying the energy and protein requirements of different patient groups, and determining whether restrictions are necessary on salt, fluids, and other substrate intake.

While NAFLD may impact over 30% of the general population, it is regrettable that there is only a limited amount of research on the impact of BCAA supplementation in NAFLD animal models and clinical trials assessing the efficacy of supplementing leucine in conjunction with other therapeutic measures. The lack of clinical studies on NAFLD patients taking BCAA-enriched supplements prevents us from determining the potential benefits of BCAA supplementation in these patients.

## 5. Conclusions

The exploration of BCAAs within the framework of liver disease has unveiled a complex interplay between metabolism, signaling pathways, disease progression, and therapeutic interventions. The intricate mechanisms underlying BCAA metabolism and signal transduction within the liver have shed light on their crucial function in maintaining liver function and homeostasis. Circulating levels of BCAAs exhibit varying patterns across distinct liver conditions. While some studies have highlighted the potential benefits of BCAAs in improving nitrogen metabolism, preserving serum albumin levels, and potentially preventing liver cancer progression, the association between BCAA intake and liver-related outcomes in certain patient populations remains inconclusive. BCAA supplements are administered orally, so the patient’s baseline hepatic functional reserve and compliance during the trial period significantly impact the outcomes. While several guidelines recommend BCAA supplementation for patients with cirrhosis, there is still no consensus on the optimal dosage, frequency, and duration, with considerable variation among numerous clinical trials. The inconsistency in these factors has escalated the heterogeneity of clinical trials, leading to an ongoing debate regarding the efficacy of BCAAs in treating liver diseases, despite decades of clinical practice. Considering the intricate nature of chronic liver diseases, future clinical trials will require more detailed patient stratification to identify the specific patient populations that may benefit most from BCAA supplementation and evaluate the impact of baseline liver function reserves on treatment outcomes. Many clinical trials are of short duration, and to establish standardized clinical practice guidelines, there is a need for more long-term clinical trials assessing the sustained effects of BCAA supplementation on liver health and disease progression. Future research directions should focus on elucidating the precise mechanisms through which BCAAs influence liver disease progression, and optimizing therapeutic strategies involving BCAAs. By bridging the gap between basic science discoveries and clinical applications, further advancements in our understanding of BCAAs and their impact on liver disease hold great promise for improving patient care and outcomes in the realm of hepatology.

## Figures and Tables

**Table 1:** Clinical trials utilizing BCAAs for the treatment of HCC.

Year	Hepatocellular Carcinoma	Duration	Study Design	Sample Size	Interventions	Frequency	Major Outcome	Ref.
1997	after curative resection	3 years	RCT	150	100 g Aminoleban^®^ EN (contains 11 g BCAAs) daily for at least 1 year	bid	• improved clinical features and laboratory data without increasing the rate of tumor recurrence	[86]
1999	after hepatic resection	1 year	RCT	44	150 g Aminoleban^®^ EN (contains 16.5 g BCAAs) daily for 12 weeks	tid	• a shorter hospital stay • quicker improvement of liver function	[92]
2004	undergoing chemoembolization	1 year	RCT	84	100 g Aminoleban^®^ EN (contains 11 g BCAAs) daily	bid	• increased serum albumin level • reduced the morbidity • improve QOL	[87]
2005	complicated with cirrhosis after hepatectomy	1 year	RCT	43	14.22 g LIVACT^®^ Granules (contains 12 g BCAAs) daily	tid	• maintained a higher serum albumin level • decreased liver fibrosis	[88]
2009	complicated with cirrhosis undergoing chemoembolization	2 weeks	RCT	56	50 g Aminoleban^®^ EN (contains 5.5 g BCAAs) daily	qd	• prevented suppression of liver function by TACE	[85]
2010	complicated with cirrhosis, underwent RFA (HCV)	1 year		49	100 g Aminoleban^®^ EN (contains 11 g BCAAs) daily	bid	• improved both nutritional state and QOL	[93]
2010	undergoing HAIC	5 weeks	RCT	23	50 g Aminoleban^®^ EN (contains 5.5 g BCAAs) at 22:00	qd	• improved energy metabolism and glucose tolerance	[101]
2010	undergoing radiotherapy	6 weeks	RCT	50	14.22 g LIVACT^®^ Granules (contains 12 g BCAAs) daily during radiotherapy	tid	• improved biochemical profiles	[98]
2010	after hepatic resection	26 weeks	RCT	96	100 g Aminoleban^®^ EN (contains 11 g BCAAs) daily	bid	• improved postoperative QOL over the long term	[94]
2011	underwent RFA	4 years	RCT	110	ACEI (perindopril; 4 mg/day) or BCAA granules (Livact; 12 g/day) or ACEI + BCAA		• ACEI + BCAA markedly inhibited the cumulative recurrence of HCC under IR conditions • neither single treatment exerted a significant inhibition	[99]
2012	complicated with cirrhosis, underwent RFA	3 months	RCT	30	50 g Aminoleban^®^ EN (contains 5.5 g BCAAs) daily after breakfast or at 22:00	qd	• improved liver functioning and Child–Pugh score	[90]
2012	after hepatic resection	26 weeks	RCT	56	14.22 g LIVACT^®^ Granules (contains 12 g BCAAs) daily	tid	• reduced early recurrence	[95]
2013	underwent local curative therapy (IR)	60 months	RCT	93	14.22 g LIVACT^®^ Granules (contains 12 g BCAAs) daily	tid	• BCAAs functioned via coordinated effects of anti-angiogenesis and IR improvement	[91]
2016	undergoing major liver resection	13 months	RCT	77	14.22 g LIVACT^®^ Granules (contains 12 g BCAAs) daily for 1 month before liver resection and 1 year after	tid	• preoperative administration of BCAA did not significantly improve the prevention of refractory ascites • prevented ascites, pleural effusion, or both • improved the metabolism of albumin	[89]
2017	underwent RFA	5 years	RCT	51	100 g Aminoleban^®^ EN (contains 11 g BCAAs) daily	bid	• relieved mental stress • reduced the risks of intrahepatic recurrence and complications	[96]
2019	(normal albumin levels and low BTRs)	10 years		78	14.22 g LIVACT^®^ Granules (contains 12 g BCAAs) daily	tid	• improved both overall survival and disease-specific survival	[100]
2020	after curative resection	4 years	RCT	156	14.22 g LIVACT^®^ Granules (contains 12 g BCAAs) daily	tid	• did not reduce the risk of recurrence • was beneficial for patients who were younger and had mildly impaired glucose tolerance	[97]

RCT, randomized controlled trial; QOL, quality of life; TACE, transcatheter arterial chemoembolization; RFA, radiofrequency ablation; HAIC, hepatic arterial infusion chemotherapy; IR, insulin resistance; BTR, BCAA-to-tyrosine ratio.

**Table 2:** Clinical trials utilizing BCAAs for the treatment of cirrhosis.

Year	Cirrhosis	Duration	Study Design	Sample Size	Interventions	Frequency	Major Outcome	Ref.
1985	cirrhosis	6 weeks	SAT	10	150 g SF-1008C (contains 18.45 g BCAAs) daily for 2 weeks	tid	• no deleterious effects on nitrogen metabolism • useful for the improvement of plasma amino acid imbalance and PEM	[82]
2001	cirrhosis	28 days	SAT	14	100 g Aminoleban^®^ (contains 11 g BCAAs) daily at 8:30 and 19:00 or at 8:30 and 22:30	bid	• late-evening BCAA supplementation was more helpful in improving protein catabolism and lipolysis	[120]
2003	compensated	3 weeks and 3 months	crossover study and RCT	24	14.22 g LIVACT^®^ Granules (contains 12 g BCAAs) daily 4g after each meal (at 8:30 AM, 12:30 PM, and 6:30 PM), or 4 g at 8:30 AM and 8 g at 11 PM	bid or tid	• nocturnal BCAA administration improved serum albumin levels, whereas daytime administration did not	[121]
2003	advanced	15 months	RCT	174	14.4 g BCAAs daily for 1 year	tid	• prevented progressive hepatic failure • improved surrogate markers and perceived health status	[108]
2004	early-stage (HCV)	2 years	RCT	65	14.22 g LIVACT^®^ Granules (contains 12 g BCAAs) daily	tid	• maintained serum albumin • improved prognosis and maintained QOL	[102]
2005	cirrhosis	7 days	RCT	26	50 g Aminoleban^®^ EN (contains 5.5 g BCAAs) daily at 22:00 or 100 g Aminoleban^®^ EN (contains 11 g BCAAs) daily at 22:00 and in the daytime	bid or tid	• LESs alone improved the energy malnutrition state and glucose intolerance to the same extent as LESs combined with divided meals	[122]
2005	decompensated	2 years	RCT	646	14.22 g LIVACT^®^ Granules (contains 12 g BCAAs) daily	tid	• improved event-free survival, serum albumin concentration, and QOL	[103]
2005	decompensated (HE, hypoalbuminemia)	6 months	RCT	281	14.22 g LIVACT^®^ Granules (contains 12 g BCAAs) or 100 g Aminoleban^®^ EN (contains 12.5 g BCAAs) daily	bid or tid	• adequate BCAAs alone improved serum albumin profiles to a similar extent as the oral nutritional supplementation	[104]
2006	decompensated (hypoalbuminemia)	2 years	RCT	622	14.22 g LIVACT^®^ Granules (contains 12 g BCAAs) daily	tid	• the risk for liver cancer was significantly reduced in the BCAA group with a BMI of 25 or higher and with an AFP level of 20 ng/mL or higher	[53]
2007	advanced (HCV)	3 months	RCT	48	6.075 g of BCAAs daily	once a day before bedtime	• long-term oral supplementation of BCAA as LESs could better improve serum albumin levels and energy metabolism compared to regular food	[105]
2008	cirrhosis	3 months	SAT	11	50 g Aminoleban^®^ EN (contains 5.5 g BCAAs) + 0.2 mg voglibose daily	qd	• the combination of α-glucosidase inhibitors with BCAA-enriched LESs showed potential for improving glucose tolerance and energy metabolism	[123]
2008	compensated (HCV)	3.5 years	RCT	40	12 g BCAAs daily for 168 weeks	tid	• BCAA may inhibit hepatic carcinogenesis in patients with compensated cirrhosis with a serum albumin level of <4.0 g/dL	[107]
2009	early stage	6 years	RCT	56	14.22 g LIVACT^®^ Granules (contains 12 g BCAAs) daily for at least 1 year	tid	• early interventional oral BCAAs might prolong the liver transplant waiting period by preserving hepatic reserve in cirrhosis	[109]
2009	decompensated and compensated (HCV)	2 years	RCT	65	14.22 g LIVACT^®^ Granules (contains 12 g BCAAs) daily	tid	• if cirrhotic patients were in the compensated stage at the entrybut with lower BTR, as for decompensated cirrhosis, oral BCAA supplementation might be effective in maintaining serum albumin levels for 2 years	[106]
2010	(a previous episode of HE)	8 weeks	RCT	21	50 g Aminoleban^®^ EN (contains 5.5 g BCAAs) at 22:00	qd	• beneficial for patients with sleep disturbance	[124]
2011	cirrhosis	6 months	SAT	17	100 g Aminoleban^®^ EN (contains 11 g BCAAs) daily at 22:00 and in the daytime	bid	• BCAA-enriched LESs could improve protein malnutrition and improve hepatic parenchymal cell mass in the early stages of cirrhosis	[125]
2011	(a previous episode of HE)	14 months	RCT	116	100 g BCAAs daily for 56 weeks	bid	• did not decrease the recurrence of HE • improved minimal HE and muscle mass	[111]
2013	compensated	3 months	RCT	37	14.22 g LIVACT^®^ Granules (contains 12 g BCAAs) daily	bid or tid	• nocturnal administration reduced the occurrence of muscle cramps in the leg but did not improve the patients’ QOL	[112]
2015	(alcoholic)			14	a single oral BCAA mixture enriched with leucine (BCAA/Leu) (7.5 g L-Leu, 3.75 g L-Ile, 3.75 g L-Val)		• impaired mTOR1 signaling and increased autophagy in skeletal muscle was acutely reversed	[117]
2019	cirrhosis	1 month	RCT	10	50 g Aminoleban^®^ EN (contains 5.5 g BCAAs) as LESs or 9.48 g LIVACT^®^ Granules (contains 8 g BCAAs) + 50 g Aminoleban^®^ EN (contains 5.5 g BCAAs) intraday or 9.48 g LIVACT^®^ Granules (contains 8 g BCAAs) intraday + 50 g Aminoleban^®^ EN (contains 5.5 g BCAAs) as LES	qd or tid	• increasing the fasting Fischer’s ratio required not only an increase in the intake of BCAAs, but also BCAA-enriched LES	[126]
2019	compensated (hypoalbuminemia)	15 days		13	50 g Aminoleban^®^ EN (contains 5.5 g BCAAs) as LES	qd	• may worsen glucose homeostasis in obese and IR cirrhosis patients	[127]
2021	(sarcopenia)	3 months	RCT	32	5.24 g BCAAs daily	qd	• improved muscle mass	[113]
2021	(sarcopenia)	6 months	RCT	106	7.2 g BCAAs daily	qd	• improved sarcopenia and prognostic markers	[114]
2022	(sarcopenia)	6 months	RCT	60	12 g BCAAs daily	bid	• did not improve muscle mass	[118]
2023	compensated (frailty)	4 months	RCT	54	100 g Aminoleban^®^ (contains 11 g BCAAs) daily	bid	• improved frailty • improved muscle mass and physical domain of QOL	[115]
2023	(HCV)		retrospective cohort study	656			• BCAA intake was not associated with liver-related outcomes in HCV-infected patients with advanced fibrosis or compensated cirrhosis	[110]
2024	(sarcopenia)	12 months	RCT	150	21.2 g BCAAs daily	bid or tid	• did not improve measures of muscle strength, mass, or performance or physical frailty	[119]
2024	cirrhosis	28 days	RCT	220	10 g BCAAs daily or programmed exercise or 10 g BCAAs daily and programmed exercise	qd	• improved quadriceps muscle quantity and quality	[116]

SAT, single-arm trial; PEM: protein–energy malnutrition; RCT, randomized controlled trial; HCV, hepatitis C virus; QOL, quality of life; HE, hepatic encephalopathy; AFP: alpha-fetoprotein; Leu, leucine; Ile: isoleucine; Val: valine; IR, insulin resistance.

## References

[1] Wu G. (2009). Amino Acids: Metabolism, Functions, and Nutrition. Amino Acids.

[2] Neinast M., Murashige D., Arany Z. (2019). Branched Chain Amino Acids. Annu. Rev. Physiol..

[3] Wolfson R.L., Chantranupong L., Saxton R.A., Shen K., Scaria S.M., Cantor J.R., Sabatini D.M. (2016). Sestrin2 Is a Leucine Sensor for the mTORC1 Pathway. Science.

[4] Neinast M.D., Jang C., Hui S., Murashige D.S., Chu Q., Morscher R.J., Li X., Zhan L., White E., Anthony T.G. (2019). Quantitative Analysis of the Whole-Body Metabolic Fate of Branched-Chain Amino Acids. Cell Metab..

[5] Fischer J.E., Baldessarini R.J. (1971). False Neurotransmitters and Hepatic Failure. Lancet.

[6] Yoshida T., Muto Y., Moriwaki H., Yamato M. (1989). Effect of Long-Term Oral Supplementation with Branched-Chain Amino Acid Granules on the Prognosis of Liver Cirrhosis. Gastroenterol. Jpn..

[7] Soeters P.B., Fischer J.E. (1976). Insulin, Glucagon, Aminoacid Imbalance, and Hepatic Encephalopathy. Lancet.

[8] Chashmniam S., Ghafourpour M., Farimani A.R., Gholami A., Ghoochani B.F.N.M. (2019). Metabolomic Biomarkers in the Diagnosis of Non-Alcoholic Fatty Liver Disease. Hepat. Mon..

[9] Lischka J., Schanzer A., Hojreh A., Ba Ssalamah A., Item C.B., de Gier C., Walleczek N.-K., Metz T.F., Jakober I., Greber-Platzer S. (2021). A Branched-Chain Amino Acid-Based Metabolic Score Can Predict Liver Fat in Children and Adolescents with Severe Obesity. Pediatr. Obes..

[10] Nezami Ranjbar M.R., Luo Y., Di Poto C., Varghese R.S., Ferrarini A., Zhang C., Sarhan N.I., Soliman H., Tadesse M.G., Ziada D.H. (2015). GC-MS Based Plasma Metabolomics for Identification of Candidate Biomarkers for Hepatocellular Carcinoma in Egyptian Cohort. PLoS ONE.

[11] Park J.G., Tak W.Y., Park S.Y., Kweon Y.O., Chung W.J., Jang B.K., Bae S.H., Lee H.J., Jang J.Y., Suk K.T. (2020). Effects of Branched-Chain Amino Acid (BCAA) Supplementation on the Progression of Advanced Liver Disease: A Korean Nationwide, Multicenter, Prospective, Observational, Cohort Study. Nutrients.

[12] Chen L., Chen Y., Wang X., Li H., Zhang H., Gong J., Shen S., Yin W., Hu H. (2015). Efficacy and Safety of Oral Branched-Chain Amino Acid Supplementation in Patients Undergoing Interventions for Hepatocellular Carcinoma: A Meta-Analysis. Nutr. J..

[13] Hayaishi S., Chung H., Kudo M., Ishikawa E., Takita M., Ueda T., Kitai S., Inoue T., Yada N., Hagiwara S. (2011). Oral Branched-Chain Amino Acid Granules Reduce the Incidence of Hepatocellular Carcinoma and Improve Event-Free Survival in Patients with Liver Cirrhosis. Dig. Dis..

[14] Brosnan J.T., Brosnan M.E. (2006). Branched-Chain Amino Acids: Enzyme and Substrate Regulation. J. Nutr..

[15] Suryawan A., Hawes J.W., Harris R.A., Shimomura Y., Jenkins A.E., Hutson S.M. (1998). A Molecular Model of Human Branched-Chain Amino Acid Metabolism123. Am. J. Clin. Nutr..

[16] Harper A.E., Miller R.H., Block K.P. (1984). Branched-Chain Amino Acid Metabolism. Annu. Rev. Nutr..

[17] Shin A.C., Fasshauer M., Filatova N., Grundell L.A., Zielinski E., Zhou J.Y., Scherer T., Lindtner C., White P.J., Lapworth A.L. (2014). Brain Insulin Lowers Circulating Bcaa Levels by Inducing Hepatic Bcaa Catabolism. Cell Metab..

[18] Everman S., Mandarino L.J., Carroll C.C., Katsanos C.S. (2015). Effects of Acute Exposure to Increased Plasma Branched-Chain Amino Acid Concentrations on Insulin-Mediated Plasma Glucose Turnover in Healthy Young Subjects. PLoS ONE.

[19] Azuma Y., Maekawa M., Kuwabara Y., Nakajima T., Taniguchi K., Kanno T. (1989). Determination of Branched-Chain Amino Acids and Tyrosine in Serum of Patients with Various Hepatic Diseases, and Its Clinical Usefulness. Clin. Chem..

[20] Mino M., Sano A., Kakazu E., Matsubara H., Kakisaka K., Kogure T., Sekine K., Aoki Y., Imamura M., Matsuda M. (2024). Differences in Branched-Chain Amino Acid to Tyrosine Ratio (BTR) among Etiologies of Chronic Liver Disease Progression Compared to Healthy Adults. J. Gastroenterol..

[21] Shimobayashi M., Hall M.N. (2014). Making New Contacts: The mTOR Network in Metabolism and Signalling Crosstalk. Nat. Rev. Mol. Cell Biol..

[22] Laplante M., Sabatini D.M. (2012). mTOR Signaling in Growth Control and Disease. Cell.

[23] Sancak Y., Peterson T.R., Shaul Y.D., Lindquist R.A., Thoreen C.C., Bar-Peled L., Sabatini D.M. (2008). The Rag GTPases Bind Raptor and Mediate Amino Acid Signaling to mTORC1. Science.

[24] Sancak Y., Bar-Peled L., Zoncu R., Markhard A.L., Nada S., Sabatini D.M. (2010). Ragulator-Rag Complex Targets mTORC1 to the Lysosomal Surface and Is Necessary for Its Activation by Amino Acids. Cell.

[25] Garami A., Zwartkruis F.J.T., Nobukuni T., Joaquin M., Roccio M., Stocker H., Kozma S.C., Hafen E., Bos J.L., Thomas G. (2003). Insulin Activation of Rheb, a Mediator of mTOR/S6K/4E-BP Signaling, Is Inhibited by TSC1 and 2. Mol. Cell.

[26] Han J.M., Jeong S.J., Park M.C., Kim G., Kwon N.H., Kim H.K., Ha S.H., Ryu S.H., Kim S. (2012). Leucyl-tRNA Synthetase Is an Intracellular Leucine Sensor for the mTORC1-Signaling Pathway. Cell.

[27] Bonfils G., Jaquenoud M., Bontron S., Ostrowicz C., Ungermann C., De Virgilio C. (2012). Leucyl-tRNA Synthetase Controls TORC1 via the EGO Complex. Mol. Cell.

[28] Yoon M.S., Son K., Arauz E., Han J.M., Kim S., Chen J. (2016). Leucyl-tRNA Synthetase Activates Vps34 in Amino Acid-Sensing mTORC1 Signaling. Cell Rep..

[29] Yuan H.-X., Xiong Y., Guan K.-L. (2013). Nutrient Sensing, Metabolism, and Cell Growth Control. Mol. Cell.

[30] Hay N., Sonenberg N. (2004). Upstream and Downstream of mTOR. Genes. Dev..

[31] Inoki K., Li Y., Xu T., Guan K.-L. (2003). Rheb GTpase Is a Direct Target of TSC2 GAP Activity and Regulates mTOR Signaling. Genes. Dev..

[32] Alessi D.R., James S.R., Downes C.P., Holmes A.B., Gaffney P.R.J., Reese C.B., Cohen P. (1997). Characterization of a 3-Phosphoinositide-Dependent Protein Kinase Which Phosphorylates and Activates Protein Kinase Bα. Curr. Biol..

[33] Alessi D.R., Andjelkovic M., Caudwell B., Cron P., Morrice N., Cohen P., Hemmings B.A. (1996). Mechanism of Activation of Protein Kinase B by Insulin and IGF-1. EMBO J..

[34] Inoki K., Li Y., Zhu T., Wu J., Guan K.-L. (2002). TSC2 Is Phosphorylated and Inhibited by Akt and Suppresses mTOR Signalling. Nat. Cell Biol..

[35] Tzatsos A., Kandror K.V. (2006). Nutrients Suppress Phosphatidylinositol 3-Kinase/Akt Signaling via Raptor-Dependent mTOR-Mediated Insulin Receptor Substrate 1 Phosphorylation. Mol. Cell Biol..

[36] Um S.H., Frigerio F., Watanabe M., Picard F., Joaquin M., Sticker M., Fumagalli S., Allegrini P.R., Kozma S.C., Auwerx J. (2004). Absence of S6K1 Protects against Age- and Diet-Induced Obesity While Enhancing Insulin Sensitivity. Nature.

[37] Hagiwara A., Nishiyama M., Ishizaki S. (2012). Branched-Chain Amino Acids Prevent Insulin-Induced Hepatic Tumor Cell Proliferation by Inducing Apoptosis through mTORC1 and mTORC2-Dependent Mechanisms. J. Cell. Physiol..

[38] Loomba R., Sanyal A.J. (2013). The Global NAFLD Epidemic. Nat. Rev. Gastroenterol. Hepatol..

[39] Rinella M.E., Lazarus J.V., Ratziu V., Francque S.M., Sanyal A.J., Kanwal F., Romero D., Abdelmalek M.F., Anstee Q.M., Arab J.P. (2023). A Multisociety Delphi Consensus Statement on New Fatty Liver Disease Nomenclature. J. Hepatol..

[40] van den Berg E.H., Flores-Guerrero J.L., Gruppen E.G., de Borst M.H., Wolak-Dinsmore J., Connelly M.A., Bakker S.J.L., Dullaart R.P.F. (2019). Non-Alcoholic Fatty Liver Disease and Risk of Incident Type 2 Diabetes: Role of Circulating Branched-Chain Amino Acids. Nutrients.

[41] Newgard C.B., An J., Bain J.R., Muehlbauer M.J., Stevens R.D., Lien L.F., Haqq A.M., Shah S.H., Arlotto M., Slentz C.A. (2009). A Branched-Chain Amino Acid-Related Metabolic Signature That Differentiates Obese and Lean Humans and Contributes to Insulin Resistance. Cell Metab..

[42] Lee C., Watkins S., Lorenzo C., Wagenknecht L., Il’yasova D., Chen Y.-D., Haffner S., Hanley A. (2016). Branched-Chain Amino Acids and Insulin Metabolism: The Insulin Resistance Atherosclerosis Study (IRAS). Diabetes Care.

[43] Sener A., Malaisse W.J. (1981). The Stimulus-Secretion Coupling of Amino Acid-Induced Insulin Release: Insulinotropic Action of Branched-Chain Amino Acids at Physiological Concentrations of Glucose and Glutamine. Eur. J. Clin. Investig..

[44] Gaggini M., Carli F., Rosso C., Buzzigoli E., Marietti M., Della Latta V., Ciociaro D., Abate M.L., Gambino R., Cassader M. (2018). Altered Amino Acid Concentrations in NAFLD: Impact of Obesity and Insulin Resistance. Hepatology.

[45] Cheng S., Wiklund P., Autio R., Borra R., Ojanen X., Xu L., Törmäkangas T., Alen M. (2015). Adipose Tissue Dysfunction and Altered Systemic Amino Acid Metabolism Are Associated with Non-Alcoholic Fatty Liver Disease. PLoS ONE.

[46] Grzych G., Vonghia L., Bout M.-A., Weyler J., Verrijken A., Dirinck E., Chevalier Curt M.J., Van Gaal L., Paumelle R., Francque S. (2020). Plasma BCAA Changes in Patients With NAFLD Are Sex Dependent. J. Clin. Endocrinol. Metab..

[47] Lagiou P., Kuper H., Stuver S.O., Tzonou A., Trichopoulos D., Adami H.-O. (2000). Role of Diabetes Mellitus in the Etiology of Hepatocellular Carcinoma. JNCI J. Natl. Cancer Inst..

[48] Shimada M., Hashimoto E., Taniai M., Hasegawa K., Okuda H., Hayashi N., Takasaki K., Ludwig J. (2002). Hepatocellular Carcinoma in Patients with Non-Alcoholic Steatohepatitis. J. Hepatol..

[49] El-serag H.B., Tran T., Everhart J.E. (2004). Diabetes Increases the Risk of Chronic Liver Disease and Hepatocellular Carcinoma. Gastroenterology.

[50] Ti H., Jc W., Wy L., Yh H., Pc L., Jh C., Fy C., Sd L. (2004). Differential Mechanism and Prognostic Impact of Diabetes Mellitus on Patients with Hepatocellular Carcinoma Undergoing Surgical and Nonsurgical Treatment. Am. J. Gastroenterol..

[51] Miuma S., Ichikawa T., Taura N., Shibata H., Takeshita S., Akiyama M., Motoyoshi Y., Ozawa E., Fujimoto M., Kawashimo H. (2009). The Level of Fasting Serum Insulin, but Not Adiponectin, Is Associated with the Prognosis of Early Stage Hepatocellular Carcinoma. Oncol. Rep..

[52] Saito K., Inoue S., Saito T., Kiso S., Ito N., Tamura S., Watanabe H., Takeda H., Misawa H., Togashi H. (2002). Augmentation Effect of Postprandial Hyperinsulinaemia on Growth of Human Hepatocellular Carcinoma. Gut.

[53] Muto Y., Sato S., Watanabe A., Moriwaki H., Suzuki K., Kato A., Kato M., Nakamura T., Higuchi K., Nishiguchi S. (2006). Overweight and Obesity Increase the Risk for Liver Cancer in Patients with Liver Cirrhosis and Long-Term Oral Supplementation with Branched-Chain Amino Acid Granules Inhibits Liver Carcinogenesis in Heavier Patients with Liver Cirrhosis. Hepatol. Res. Off. J. Jpn. Soc. Hepatol..

[54] Yoshiji H., Noguchi R., Kitade M., Kaji K., Ikenaka Y., Namisaki T., Yoshii J., Yanase K., Yamazaki M., Tsujimoto T. (2009). Branched-Chain Amino Acids Suppress Insulin-Resistance-Based Hepatocarcinogenesis in Obese Diabetic Rats. J. Gastroenterol..

[55] Miuma S., Ichikawa T., Arima K., Takeshita S., Muraoka T., Matsuzaki T., Ootani M., Shibata H., Akiyama M., Ozawa E. (2012). Branched-Chain Amino Acid Deficiency Stabilizes Insulin-Induced Vascular Endothelial Growth Factor mRNA in Hepatocellular Carcinoma Cells. J. Cell Biochem..

[56] Nishitani S., Horie M., Ishizaki S., Yano H. (2013). Branched Chain Amino Acid Suppresses Hepatocellular Cancer Stem Cells through the Activation of Mammalian Target of Rapamycin. PLoS ONE.

[57] Hiraoka A., Kato M., Marui K., Murakami T., Onishi K., Adachi T., Matsuoka J., Ueki H., Yoshino T., Tsuruta M. (2021). Easy Clinical Predictor for Low BCAA to Tyrosine Ratio in Chronic Liver Disease Patients with Hepatocellular Carcinoma: Usefulness of ALBI Score as Nutritional Prognostic Marker. Cancer Med..

[58] Ghanem S.E., Abdel-Samiee M., El-Said H., Youssef M.I., ElZohry H.A., Abdelsameea E., Moaz I., Abdelwahab S.F., Elaskary S.A., Zaher E.M. (2022). Evaluation of Amino Acids Profile as Non-Invasive Biomarkers of Hepatocellular Carcinoma in Egyptians. Trop. Med. Infect. Dis..

[59] Buchard B., Teilhet C., Abeywickrama Samarakoon N., Massoulier S., Joubert-Zakeyh J., Blouin C., Reynes C., Sabatier R., Biesse-Martin A.-S., Vasson M.-P. (2021). Two Metabolomics Phenotypes of Human Hepatocellular Carcinoma in Non-Alcoholic Fatty Liver Disease According to Fibrosis Severity. Metabolites.

[60] Ericksen R.E., Lim S.L., McDonnell E., Shuen W.H., Vadiveloo M., White P.J., Ding Z., Kwok R., Lee P., Radda G.K. (2019). Loss of BCAA Catabolism during Carcinogenesis Enhances mTORC1 Activity and Promotes Tumor Development and Progression. Cell Metab..

[61] Higashi T., Hayashi H., Kaida T., Arima K., Takeyama H., Taki K., Izumi D., Tokunaga R., Kosumi K., Nakagawa S. (2015). Prognostic Impact of Visceral Fat Amount and Branched-Chain Amino Acids (BCAA) in Hepatocellular Carcinoma. Ann. Surg. Oncol..

[62] Morgan M.Y., Milsom J.P., Sherlock S. (1978). Plasma Ratio of Valine, Leucine and Isoleucine to Phenylalanine and Tyrosine in Liver Disease. Gut.

[63] Tajika M., Kato M., Mohri H., Miwa Y., Kato T., Ohnishi H., Moriwaki H. (2002). Prognostic Value of Energy Metabolism in Patients with Viral Liver Cirrhosis. Nutrition.

[64] Yamato M., Muto Y., Yoshida T., Kato M., Moriwaki H. (1995). Clearance Rate of Plasma Branched-Chain Amino Acids Correlates Significantly with Blood Ammonia Level in Patients with Liver Cirrhosis. Int. Hepatol. Commun..

[65] Kato M., Miwa Y., Tajika M., Hiraoka T., Muto Y., Moriwaki H. (1998). Preferential Use of Branched-Chain Amino Acids as an Energy Substrate in Patients with Liver Cirrhosis. Intern. Med..

[66] Grattagliano I., Russmann S., Diogo C., Bonfrate L., Oliveira P., Wang D., Portincasa P. (2011). Mitochondria in Chronic Liver Disease. Curr. Drug Targets.

[67] James J.H. (2002). Branched Chain Amino Acids in Heptatic Encephalopathy. Am. J. Surg..

[68] Fischer J.E., Rosen H.M., Ebeid A.M., James J.H., Keane J.M., Soeters P.B. (1976). The Effect of Normalization of Plasma Amino Acids on Hepatic Encephalopathy in Man. Surgery.

[69] Kakazu E., Kanno N., Ueno Y., Shimosegawa T. (2007). Extracellular Branched-Chain Amino Acids, Especially Valine, Regulate Maturation and Function of Monocyte-Derived Dendritic Cells. J. Immunol..

[70] Kakazu E., Ueno Y., Kondo Y., Fukushima K., Shiina M., Inoue J., Tamai K., Ninomiya M., Shimosegawa T. (2009). Branched Chain Amino Acids Enhance the Maturation and Function of Myeloid Dendritic Cells Ex Vivo in Patients with Advanced Cirrhosis. Hepatology.

[71] Kawaguchi T., Torimura T., Takata A., Satomi S., Sata M. (2012). Valine, a Branched-Chain Amino Acid, Reduced HCV Viral Load and Led to Eradication of HCV by Interferon Therapy in a Decompensated Cirrhotic Patient. Case Rep. Gastroenterol..

[72] Ishida H., Kato T., Takehana K., Tatsumi T., Hosui A., Nawa T., Kodama T., Shimizu S., Hikita H., Hiramatsu N. (2013). Valine, the Branched-Chain Amino Acid, Suppresses Hepatitis C Virus RNA Replication but Promotes Infectious Particle Formation. Biochem. Biophys. Res. Commun..

[73] Honda M., Takehana K., Sakai A., Tagata Y., Shirasaki T., Nishitani S., Muramatsu T., Yamashita T., Nakamoto Y., Mizukoshi E. (2011). Malnutrition Impairs Interferon Signaling through mTOR and FoxO Pathways in Patients with Chronic Hepatitis C. Gastroenterology.

[74] Nagao Y., Kawaguchi T., Ide T., Sata M. (2012). Effect of Branched-Chain Amino Acid-Enriched Nutritional Supplementation on Interferon Therapy in Japanese Patients with Chronic Hepatitis C Virus Infection: A Retrospective Study. Virol. J..

[75] Honda T., Fukuda Y., Nakano I., Katano Y., Goto H., Nagasaki M., Sato Y., Murakami T., Shimomura Y. (2004). Effects of Liver Failure on Branched-Chain Alpha-Keto Acid Dehydrogenase Complex in Rat Liver and Muscle: Comparison between Acute and Chronic Liver Failure. J. Hepatol..

[76] Kitagawa T., Yokoyama Y., Kokuryo T., Nagino M. (2013). Protective Effects of Branched-Chain Amino Acids on Hepatic Ischemia-Reperfusion-Induced Liver Injury in Rats: A Direct Attenuation of Kupffer Cell Activation. Am. J. Physiol.-Gastrointest. Liver Physiol..

[77] Deshpande G., Adachi N., Liu K., Motoki A., Mitsuyo T., Nagaro T., Arai T. (2007). Recovery of Brain Dopamine Metabolism by Branched-Chain Amino Acids in Rats with Acute Hepatic Failure. J. Neurosurg. Anesthesiol..

[78] Kuwahata M., Kuramoto Y., Tomoe Y., Sugata E., Segawa H., Ito M., Oka T., Miyamoto K. (2004). Posttranscriptional Regulation of Albumin Gene Expression by Branched-Chain Amino Acids in Rats with Acute Liver Injury. Biochim. Et Biophys. Acta (BBA)—Mol. Basis Dis..

[79] Rigotti P., Peters J.C., Tranberg K.-G., Fischer J.E. (1986). Effects of Amino Acid Infusions on Liver Regeneration after Partial Hepatectomy in the Rat. JPEN. J. Parenter. Enter. Nutr..

[80] Holecek M., Simek J., Kruf M., Zadák Z. (1985). Effect of Branched Chain Amino Acids on Liver Regeneration after Partial Hepatectomy. Physiol. Bohemoslov..

[81] Holecek M., Simek J., Palicka V., Zadák Z. (1991). Effect of Glucose and Branched Chain Amino Acid (BCAA) Infusion on Onset of Liver Regeneration and Plasma Amino Acid Pattern in Partially Hepatectomized Rats. J. Hepatol..

[82] Okita M., Watanabe A., Nagashima H. (1985). Nutritional Treatment of Liver Cirrhosis by Branched-Chain Amino Acid-Enriched Nutrient Mixture. J. Nutr. Sci. Vitaminol..

[83] American Association for the Study of Liver Diseases, European Association for the Study of the Liver (2014). Hepatic Encephalopathy in Chronic Liver Disease: 2014 Practice Guideline by the European Association for the Study of the Liver and the American Association for the Study of Liver Diseases. J. Hepatol..

[84] Plauth M., Bernal W., Dasarathy S., Merli M., Plank L.D., Schütz T., Bischoff S.C. (2019). ESPEN Guideline on Clinical Nutrition in Liver Disease. Clin. Nutr..

[85] Takeshita S., Ichikawa T., Nakao K., Miyaaki H., Shibata H., Matsuzaki T., Muraoka T., Honda T., Otani M., Akiyama M. (2009). A Snack Enriched with Oral Branched-Chain Amino Acids Prevents a Fall in Albumin in Patients with Liver Cirrhosis Undergoing Chemoembolization for Hepatocellular Carcinoma. Nutr. Res..

[86] San-in Group of Liver Surgery (1997). Long-Term Oral Administration of Branched Chain Amino Acids after Curative Resection of Hepatocellular Carcinoma: A Prospective Randomized Trial. The San-in Group of Liver Surgery. Br. J. Surg..

[87] Poon R.T.-P., Yu W.-C., Fan S.-T., Wong J. (2004). Long-Term Oral Branched Chain Amino Acids in Patients Undergoing Chemoembolization for Hepatocellular Carcinoma: A Randomized Trial. Aliment. Pharmacol. Ther..

[88] Togo S., Tanaka K., Morioka D., Sugita M., Ueda M., Miura Y., Kubota T., Nagano Y., Matsuo K., Endo I. (2005). Usefulness of Granular BCAA after Hepatectomy for Liver Cancer Complicated with Liver Cirrhosis. Nutrition.

[89] Kikuchi Y., Hiroshima Y., Matsuo K., Kawaguchi D., Murakami T., Yabushita Y., Endo I., Taguri M., Koda K., Tanaka K. (2016). A Randomized Clinical Trial of Preoperative Administration of Branched-Chain Amino Acids to Prevent Postoperative Ascites in Patients with Liver Resection for Hepatocellular Carcinoma. Ann. Surg. Oncol..

[90] Morihara D., Iwata K., Hanano T., Kunimoto H., Kuno S., Fukunaga A., Yotsumoto K., Takata K., Tanaka T., Sakurai K. (2012). Late-Evening Snack with Branched-Chain Amino Acids Improves Liver Function after Radiofrequency Ablation for Hepatocellular Carcinoma. Hepatol. Res..

[91] Yoshiji H., Noguchi R., Namisaki T., Moriya K., Kitade M., Aihara Y., Douhara A., Yamao J., Fujimoto M., Toyohara M. (2013). Branched-Chain Amino Acids Suppress the Cumulative Recurrence of Hepatocellular Carcinoma under Conditions of Insulin-Resistance. Oncol. Rep..

[92] Meng W.C., Leung K.L., Ho R.L., Leung T.W., Lau W.Y. (1999). Prospective Randomized Control Study on the Effect of Branched-Chain Amino Acids in Patients with Liver Resection for Hepatocellular Carcinoma. Aust. N. Z. J. Surg..

[93] Kuroda H., Ushio A., Miyamoto Y., Sawara K., Oikawa K., Kasai K., Endo R., Takikawa Y., Kato A., Suzuki K. (2010). Effects of Branched-Chain Amino Acid-Enriched Nutrient for Patients with Hepatocellular Carcinoma Following Radiofrequency Ablation: A One-Year Prospective Trial. J. Gastroenterol. Hepatol..

[94] Okabayashi T., Iyoki M., Sugimoto T., Kobayashi M., Hanazaki K. (2011). Oral Supplementation with Carbohydrate- and Branched-Chain Amino Acid-Enriched Nutrients Improves Postoperative Quality of Life in Patients Undergoing Hepatic Resection. Amino Acids.

[95] Ichikawa K., Okabayashi T., Maeda H., Namikawa T., Iiyama T., Sugimoto T., Kobayashi M., Mimura T., Hanazaki K. (2013). Oral Supplementation of Branched-Chain Amino Acids Reduces Early Recurrence after Hepatic Resection in Patients with Hepatocellular Carcinoma: A Prospective Study. Surg. Today.

[96] Nojiri S., Fujiwara K., Shinkai N., Iio E., Joh T. (2017). Effects of Branched-Chain Amino Acid Supplementation after Radiofrequency Ablation for Hepatocellular Carcinoma: A Randomized Trial. Nutrition.

[97] Hachiya H., Aoki T., Iso Y., Shimizu T., Tago K., Park K.H., Sakuraoka Y., Shiraki T., Mori S., Kubota K. (2020). Effects of Branched-Chain Amino Acids on Postoperative Tumor Recurrence in Patients Undergoing Curative Resection for Hepatocellular Carcinoma: A Randomized Clinical Trial. J. Hepatobiliary Pancreat. Sci..

[98] Lee I.J., Seong J., Bae J.I., You S.H., Rhee Y., Lee J.H. (2011). Effect of Oral Supplementation with Branched-Chain Amino Acid (BCAA) during Radiotherapy in Patients with Hepatocellular Carcinoma: A Double-Blind Randomized Study. Cancer Res. Treat..

[99] Yoshiji H., Noguchi R., Ikenaka Y., Kaji K., Aihara Y., Yamazaki M., Yamao J., Toyohara M., Mitoro A., Sawai M. (2011). Combination of Branched-Chain Amino Acids and Angiotensin-Converting Enzyme Inhibitor Suppresses the Cumulative Recurrence of Hepatocellular Carcinoma: A Randomized Control Trial. Oncol. Rep..

[100] Tada T., Kumada T., Toyoda H., Yasuda S., Koyabu T., Nakashima M. (2019). Impact of Branched-Chain Amino Acid Granule Therapy in Patients with Hepatocellular Carcinoma Who Have Normal Albumin Levels and Low Branched-Chain Amino Acid to Tyrosine Ratios. Nutr. Cancer.

[101] Harima Y., Yamasaki T., Hamabe S., Saeki I., Okita K., Terai S., Sakaida I. (2010). Effect of a Late Evening Snack Using Branched-Chain Amino Acid-Enriched Nutrients in Patients Undergoing Hepatic Arterial Infusion Chemotherapy for Advanced Hepatocellular Carcinoma. Hepatol. Res..

[102] Nishiguchi S., Habu D. (2004). Effect of Oral Supplementation with Branched-Chain Amino Acid Granules in the Early Stage of Cirrhosis. Hepatol. Res..

[103] Muto Y., Sato S., Watanabe A., Moriwaki H., Suzuki K., Kato A., Kato M., Nakamura T., Higuchi K., Nishiguchi S. (2005). Effects of Oral Branched-Chain Amino Acid Granules on Event-Free Survival in Patients with Liver Cirrhosis. Clin. Gastroenterol. Hepatol..

[104] Sato S., Watanabe A., Muto Y., Suzuki K., Kato A., Moriwaki H., Kato M., Nakamura T. (2005). LIV-EN Study Group Clinical Comparison of Branched-Chain Amino Acid (l-Leucine, l-Isoleucine, l-Valine) Granules and Oral Nutrition for Hepatic Insufficiency in Patients with Decompensated Liver Cirrhosis (LIV-EN Study). Hepatol. Res..

[105] Nakaya Y., Okita K., Suzuki K., Moriwaki H., Kato A., Miwa Y., Shiraishi K., Okuda H., Onji M., Kanazawa H. (2007). BCAA-Enriched Snack Improves Nutritional State of Cirrhosis. Nutrition.

[106] Habu D., Nishiguchi S., Nakatani S., Lee C., Enomoto M., Tamori A., Takeda T., Ohfuji S., Fukushima W., Tanaka T. (2009). Comparison of the Effect of BCAA Granules on between Decompensated and Compensated Cirrhosis. Hepatogastroenterology.

[107] Kobayashi M., Ikeda K., Arase Y., Suzuki Y., Suzuki F., Akuta N., Hosaka T., Murashima N., Saitoh S., Someya T. (2008). Inhibitory Effect of Branched-Chain Amino Acid Granules on Progression of Compensated Liver Cirrhosis Due to Hepatitis C Virus. J. Gastroenterol..

[108] Marchesini G., Bianchi G., Merli M., Amodio P., Panella C., Loguercio C., Rossi Fanelli F., Abbiati R. (2003). Nutritional Supplementation with Branched-Chain Amino Acids in Advanced Cirrhosis: A Double-Blind, Randomized Trial. Gastroenterology.

[109] Kawamura E., Habu D., Morikawa H., Enomoto M., Kawabe J., Tamori A., Sakaguchi H., Saeki S., Kawada N., Shiomi S. (2009). A Randomized Pilot Trial of Oral Branched-Chain Amino Acids in Early Cirrhosis: Validation Using Prognostic Markers for Pre-Liver Transplant Status. Liver Transpl..

[110] Yu L., Paski S.C., Dodge J., Bambha K., Biggins S.W., Ioannou G.N. (2023). Effect of Dietary Branched Chain Amino Acids on Liver Related Mortality: Results from a Large Cohort of North American Patients with Advanced HCV Infection. PLoS ONE.

[111] Les I., Doval E., García-Martínez R., Planas M., Cárdenas G., Gómez P., Flavià M., Jacas C., Mínguez B., Vergara M. (2011). Effects of Branched-Chain Amino Acids Supplementation in Patients with Cirrhosis and a Previous Episode of Hepatic Encephalopathy: A Randomized Study. Am. J. Gastroenterol..

[112] Hidaka H., Nakazawa T., Kutsukake S., Yamazaki Y., Aoki I., Nakano S., Asaba N., Minamino T., Takada J., Tanaka Y. (2013). The Efficacy of Nocturnal Administration of Branched-Chain Amino Acid Granules to Improve Quality of Life in Patients with Cirrhosis. J. Gastroenterol..

[113] Hernández-Conde M., Llop E., Gómez-Pimpollo L., Fernández Carrillo C., Rodríguez L., Van Den Brule E., Perelló C., López-Gómez M., Abad J., Martínez-Porras J.L. (2021). Adding Branched-Chain Amino Acids to an Enhanced Standard-of-Care Treatment Improves Muscle Mass of Cirrhotic Patients With Sarcopenia: A Placebo-Controlled Trial. Am. J. Gastroenterol..

[114] Singh Tejavath A., Mathur A., Nathiya D., Singh P., Raj P., Suman S., Mundada P.R., Atif S., Rai R.R., Tomar B.S. (2021). Impact of Branched Chain Amino Acid on Muscle Mass, Muscle Strength, Physical Performance, Combined Survival, and Maintenance of Liver Function Changes in Laboratory and Prognostic Markers on Sarcopenic Patients With Liver Cirrhosis (BCAAS Study): A Randomized Clinical Trial. Front. Nutr..

[115] Siramolpiwat S., Limthanetkul N., Pornthisarn B., Vilaichone R.-K., Chonprasertsuk S., Bhanthumkomol P., Nunanan P., Issariyakulkarn N. (2023). Branched-Chain Amino Acids Supplementation Improves Liver Frailty Index in Frail Compensated Cirrhotic Patients: A Randomized Controlled Trial. BMC Gastroenterol..

[116] Sobhy E., Kamal M.M., Saad Y., Saleh D.A., Elgohary R., Hassan M.S. (2024). Effect of Branched-Chain Amino Acid Supplementation and Exercise on Quadriceps Muscle Quantity and Quality in Patients with Cirrhosis as Assessed by Ultrasonography: A Randomized Controlled Trial. Clin. Nutr. ESPEN.

[117] Tsien C., Davuluri G., Singh D., Allawy A., Ten Have G.A.M., Thapaliya S., Schulze J.M., Barnes D., McCullough A.J., Engelen M.P.K.J. (2015). Metabolic and Molecular Responses to Leucine-Enriched Branched Chain Amino Acid Supplementation in the Skeletal Muscle of Alcoholic Cirrhosis. Hepatology.

[118] Mohta S., Anand A., Sharma S., Qamar S., Agarwal S., Gunjan D., Singh N., Madhusudhan K.S., Pandey R.M., Saraya A. (2022). Randomised Clinical Trial: Effect of Adding Branched Chain Amino Acids to Exercise and Standard-of-Care on Muscle Mass in Cirrhotic Patients with Sarcopenia. Hepatol. Int..

[119] Hey P., Hoermann R., Sinclair M., Chapman B., Testro A., Apostolov R., Angus P., Gow P. (2024). Branched-Chain Amino Acid Supplementation Does Not Improve Measures of Sarcopenia in Cirrhosis: Results of a Randomised Controlled Trial. Aliment. Pharmacol. Ther..

[120] Yamauchi M., Takeda K., Sakamoto K., Ohata M., Toda G. (2001). Effect of Oral Branched Chain Amino Acid Supplementation in the Late Evening on the Nutritional State of Patients with Liver Cirrhosis. Hepatol. Res..

[121] Fukushima H., Miwa Y., Ida E., Kuriyama S., Toda K., Shimomura Y., Sugiyama A., Sugihara J., Tomita E., Moriwaki H. (2003). Nocturnal Branched-Chain Amino Acid Administration Improves Protein Metabolism in Patients with Liver Cirrhosis: Comparison with Daytime Administration. J. Parenter. Enter. Nutr..

[122] Tsuchiya M., Sakaida I., Okamoto M., Okita K. (2005). The Effect of a Late Evening Snack in Patients with Liver Cirrhosis. Hepatol. Res..

[123] Korenaga K., Korenaga M., Uchida K., Yamasaki T., Sakaida I. (2008). Effects of a Late Evening Snack Combined with Alpha-Glucosidase Inhibitor on Liver Cirrhosis. Hepatol. Res..

[124] Ichikawa T., Naota T., Miyaaki H., Miuma S., Isomoto H., Takeshima F., Nakao K. (2010). Effect of an Oral Branched Chain Amino Acid-Enriched Snack in Cirrhotic Patients with Sleep Disturbance. Hepatol. Res..

[125] Koreeda C., Seki T., Okazaki K., Ha-Kawa S.K., Sawada S. (2011). Effects of Late Evening Snack Including Branched-Chain Amino Acid on the Function of Hepatic Parenchymal Cells in Patients with Liver Cirrhosis. Hepatol. Res..

[126] Maki H., Yamanaka-Okumura H., Katayama T., Ozawa Y., Hosoda A., Kurata N., Amemiya F. (2019). Late Evening Snacks with Branched-Chain Amino Acids Improve the Fischer Ratio with Patients Liver Cirrhosis at Fasting in the next Morning. Clin. Nutr. ESPEN.

[127] Nakanishi K., Namisaki T., Mashitani T., Kaji K., Ozaki K., Saikawa S., Sato S., Inoue T., Sawada Y., Kitagawa K. (2019). Late-Evening Snack with Branched-Chain Amino Acid-Enriched Nutrients Does Not Always Inhibit Overt Diabetes in Patients with Cirrhosis: A Pilot Study. Nutrients.

